# Nutritional Profiles of Four Promising Wild Edible Plants Commonly Consumed by the Semai in Malaysia

**DOI:** 10.1016/j.cdnut.2023.100054

**Published:** 2023-02-23

**Authors:** Rachel Thomas Tharmabalan

**Affiliations:** 1 School of Ethnic Studies, Bangi, Malaysia; 2 Division of Natural Resource Economics, Graduate School of Agriculture, Kyoto, Japan

**Keywords:** Semai, wild edible plants, food and nutrition security, food systems, Malaysia

## Abstract

**Background:**

An essential dietary strategy to address the rapidly increasing risk of the double burden of malnutrition among indigenous populations around the world is to improve nutritional and food diversity utilizing varieties of traditional plant-based foods.

**Objectives:**

The objective of this research was to identify wild edible plants (WEPs) frequently consumed by the Semai and analyze their proximate and mineral composition to improve the adequacy of the local population’s nutritional intake.

**Methods:**

This study was conducted among 24 informants from 3 Semai settlements utilizing semistructured, ethnobotanical appraisal methods, proximate, and mineral analysis.

**Results:**

This research first documents the common names, ethnobotanical names, and uses of 4 WEPs commonly consumed by the Semai: Sayur manis/pucuk manis [*Sauropus androgynus* (L.) Merr.], Pucuk ubi (*Manihot esculenta Crantz),* Saya/aying (*Strobilanthes crispa* Blume)*,* and snegoh [*Diplazium esculentum* (Retz.) Sw.]. The nutritional content ranged from 3.2 to 7.7 g/100 g (ash), 2.9 to 7.2 g/100 g (protein), 1.5 to 6.2 g/100 g (carbohydrate). The mineral analysis showed that these plants contain considerable calcium, iron, potassium, and magnesium content, ranging from 176 to 243 mg/100 g, 0.7 to 2.8 mg/100 g, 295 to 527 mg/100 g, 32 to 97 mg/100 g. A comparative analysis was done with commercial market produce: *Ipomoea aquatica, Brassica rapa subsp. Chinensis* and *Brassica oleracea var. alboglabra*. The nutrient content for the 3 produce ranged from 1.2 to 2.6 g/100 g (protein), 2.18 to 4.67 g/100 g (carbohydrate), and 0.59 to 1.67 mg/100 g (iron). The results showed that *Manihot esculenta* had the highest carbohydrate, calcium, potassium, and magnesium content, whereas the highest ash and protein content was found in *Diplazium esculentum.*

**Conclusions:**

These findings show that these WEPs had higher nutritional and mineral concentrations than select market produce and can be used to strengthen food and nutrition security among the Semai. However, additional information regarding antinutrient, toxic compounds, methods of preparation, and consumption is required to determine how much they contribute to nutritional outcomes before these vegetables may be adopted as new crops. *Curr Dev Nutr* 2023;x:xx.

## Introduction

Food has long been vital to human biology and culture, supplying energy and nutrients. For millions of years, obtaining sustenance from the wild was inextricably linked to humans [1]. Tribal cultures and nontribal societies living in rural and semiurban environments have long practiced the tradition of consuming wild edible plants (WEPs) as food and medicine.

WEPs are plant species that are neither farmed or domesticated but are edible and can be found in various natural habitats [2]. WEPs are collected from a variety of environments around the world, including forests, cultivable areas, and even anthropogenically damaged zones like roadsides and wastelands, by various traditions. Research has shown that WEPs provide essential micronutrients and macronutrients needed by the body and act as functional food and are used by the rural populace to treat various ailments, sickness, and diseases. Often, WEPs have an enhanced nutritional quality compared to domestic varieties [3,4].

The Orang Asli, also known as the aboriginal peoples of peninsular Malaysia, who are still living in jungles or forest-fringed areas, are Malaysia’s primary consumers. The Orang Asli are made up of 18 different ethnic groups, which have combined to form the Negrito, Senoi, and Proto-Malay tribes. The Semai, who are a subgroup of the Senoi, are the subject of this research as they are the largest of the Senoi group and 1 of the Peninsula’s main indigenous ethnic groups. Since the Orang Asli arrived from Southern Thailand 4500 y ago, they have been utilizing these wild edibles; as such, they possess an ancient wealth of knowledge on indigenous plants and their environment [5]. In Malaysia, 20.7% of children under the age of 5 are stunted, 11.5% are wasting, and 12.7% of children aged 5 to 19 are obese [6]. Additionally, the WHO [7] recently announced that among the adult population, Malaysia has the most significant incidence of obesity in Asia, with 64% of men and 65% of women classified as either overweight or obese. The coexistence of undernutrition and overnutrition in the same population over the course of a person's life is known as the double burden of malnutrition (DBM) [8]. DBM is pronounced particularly among Malaysians due to the lack of essential micronutrient deficiency, also known as hidden hunger, which affects both developing and developed countries. According to Kennedy [9], the majority of individuals in emerging nations do not consume sufficient calories and micronutrients from their diets to meet their dietary needs.

Malaysia is one of the megadiverse countries in the world as it is home to a large proportion of endemic species and the majority of Earth’s biodiversity [10]. However, it has been reported by Humphrey [11] that deforestation is rampant in permanent forest areas around Peninsular Malaysia, which not only accelerates the loss of biodiverse flora and fauna but also impacts the lives of the Orang Aslis living around nearby surroundings. According to the Ministry of Agriculture [12], only 300 indigenous types of vegetable plants have been used as food out of the 15,000 species available. On the nutritional front, there is limited research in assessing the nutritional value of these WEPs consumed by the Orang Asli, though there has been a resurgence of interest in the significance of these plants in sustaining indigenous communities worldwide [13]. In contrast, the corpus of knowledge is more sizable on “ulam” (type of Malay traditional salad that is made from fresh vegetables, fruits, and leaves and is usually consumed raw or after steeped in hot water), which is consumed by the dominant population [14,15].

Hence, it is imperative to identify and document these WEPs found in their native environment, their sociocultural roles in achieving nutritional security by improving micronutrient consumption in individuals, as well as within the broader population. Boosting the intake of WEPs, which are valuable sources of energy and micronutrients, could be used as a policy to address specific micronutrient deficiencies among rural communities. As such, the current investigation was carried out in order to ascertain the nutritional content and mineral composition of these WEPs that the Semai of the Orang Asli people consume. These native food plants will be discussed in terms of their traditional uses and ethnobotanical significance.

## Methods

### Study area

**FIGURE 1:**
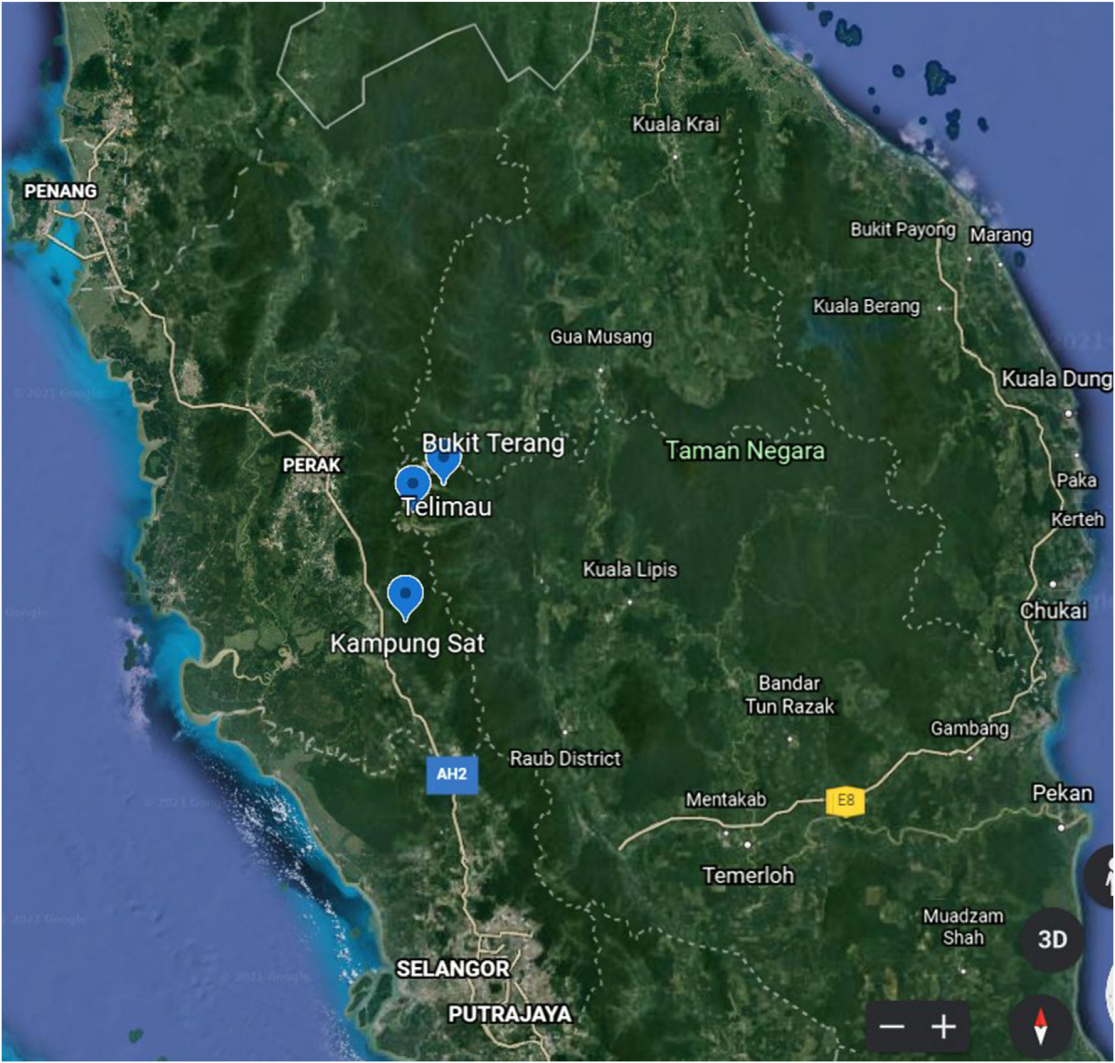
Map showing the location of Telimau, Bukit Terang, and Kampung Sat [16].

**FIGURE 2:**
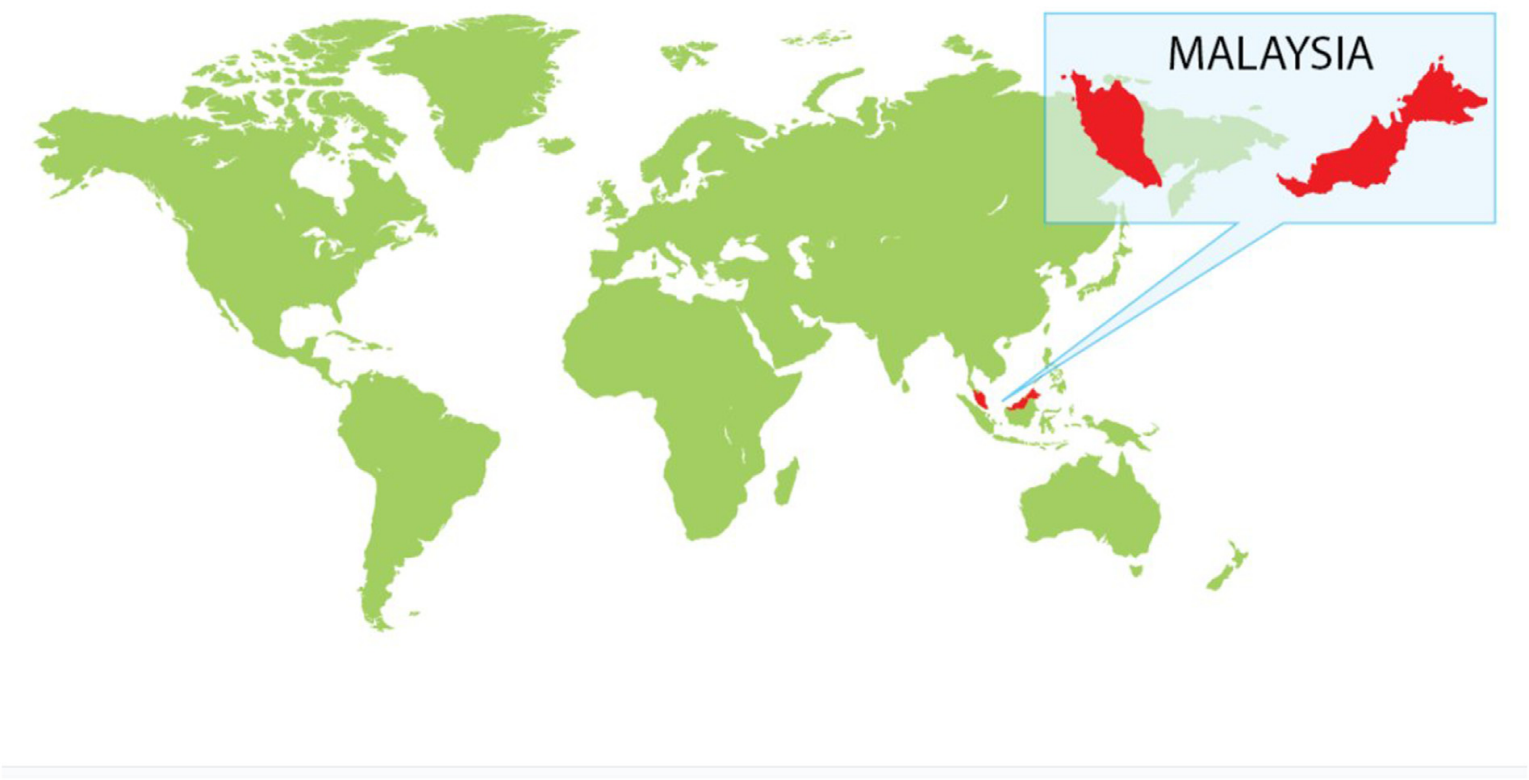
Inlaid map of Malaysia (MAFTA) [17].

Figure 1 shows the area of study, which includes Telimau, Bukit Terang, and Kampung Sat [16], whereas Figure 2 shows an inlaid map of the world location [17]. Telimau is located in Cameron Highlands at an altitude of 3349 feet and is considered an easily accessible village. Bukit Terang is considered to be a forest-fringed settlement, and Kampung Sat is considered to be a remote Semai village. Kampung Sat was chosen because it was located further into the jungle than other Semai towns, resulting in fewer adverse environmental effects.

Semistructured interviews and ethnobotanical assessment methodologies were used to document the usage of WEPs among the communities from 2017 to 2018 after obtaining permission from the headman of each village [18]. These WEPs can be harvested all year round. There were 8 informants from each village, making up 24 informants. All participants received information about the study before it began, and it was made clear to them that they could opt out at any moment. The researcher also emphasized getting permission before using any data, participant photos, or interview snippets that might end up in the final result. Additionally, the Society of Ethnobiology’s Code of Ethics [19] was followed, ensuring that the various rights noted in this study report are acknowledged, respected, and adhered to.

Three samples of fresh WEPs were harvested from each settlement, and pictures of their locations were immediately taken. The plant specimens were then packed in food-grade plastic bags and transported on the same day at chilled temperature using cold packs to the herbarium at Universiti Kebangsaan Malaysia for identification by a plant taxonomist. Along with images illustrating their morphological traits, pertinent data was documented, including their location, general habitat information, and the names of nearby plants. The plant species that were chosen for nutritional and mineral analysis were then arranged according to their species, local name, family name, the part of the plant that was used, the way it was consumed, and any potential therapeutic qualities and were taken to a nutritional laboratory for analysis immediately after to help preserve the nutrient and mineral composition of the plant samples.

### Sample collection

The edible piece was then cleaned with tap water and rinsed with distilled water whereas the inedible portion was discarded. At ambient temperature (∼27°C), the remaining moisture on the leaves evaporated. The raw samples were dried at 60°C for an entire night to achieve constant weight. To create a homogenized powder sample, the dry material was ground into powder and stored in air-tight containers at room temperature for further analysis.

Results from 3 local commercial vegetables that are frequently consumed among Malaysians were included in this research to allow for proximate and mineral comparative studies. These 3 vegetables are Ipomoea aquatica (Kangkung), Brassica rapa subsp. chinensis (Pak Choy) and Brassica oleracea var. Alboglabra (Kai Lan) and can be easily bought in local markets.

### Proximate analysis

While nitrogen was evaluated using the Kjeldahl method, and the percentage of nitrogen was converted to crude protein by multiplying by 6.25; moisture, ash, and crude fiber were measured using the official methods of the AOAC [20]. The Soxhlet technique was used to calculate the crude fat. By subtracting the gross sum of the percentages of dietary fiber, total fat, crude protein, moisture, and ash from 100, the amount of carbohydrates was calculated. All sample analyses were performed in triplicate.

### Mineral analysis

Four common minerals were identified using the atomic absorption spectrophotometric method specified by AOAC [20], including magnesium (Mg), potassium (K), iron (Fe), and calcium (Ca). Wet ashing was used to apply 12 mL of a concentrated oxi-acidic solution of perchloric acid, HClO_4_, and nitric acid, HNO_3_ (4:1). An absorbance (nm) compared with standard solution concentration (g/mL) graph was constructed with known concentrations in order to assess the mineral content under ideal circumstances. Average values were derived based on triplicate readings. The amount of each element in 1 g of the sample was determined using the formula below:

Amount of Element, ppm (μg/g) = (μg/mL) × F/ g sample where, F = (mL original dilution × mL final dilution)/mL aliquots if original 100 mL is diluted. The data were expressed as mg/100 g.

### Statistical analysis

Using the Statistical Package for the Social Sciences version 16.0 software, 1-factor ANOVA was used to analyze the variation in these means at a *P* value of 0.05.

## Results and Discussion

### Ethnobotanical observations

**FIGURE 3:**
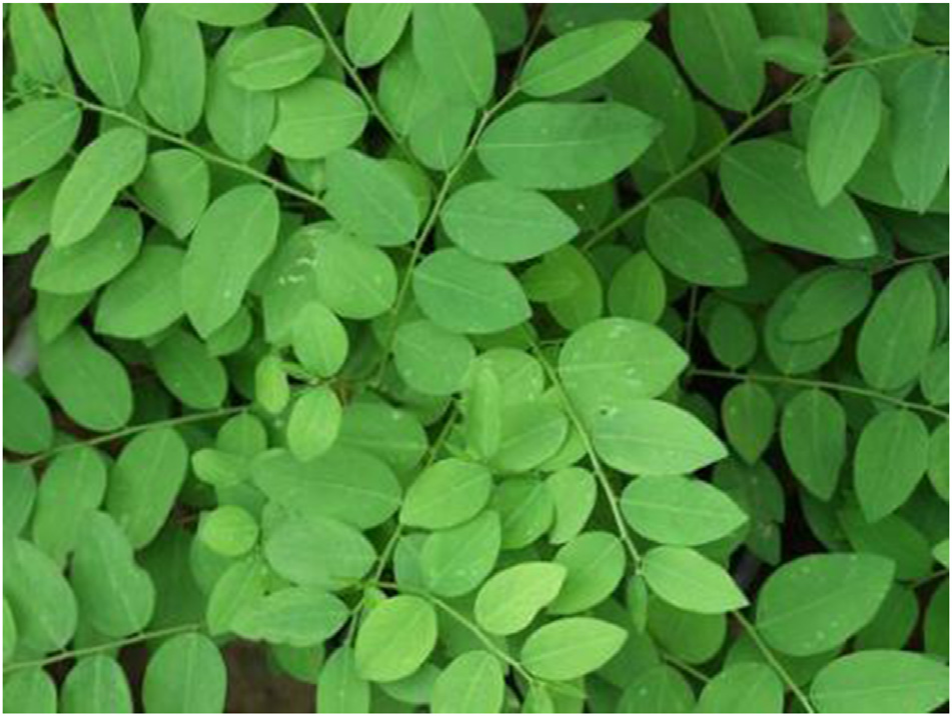
*Sauropus androgynus* (L.) Merr. (Rachel Thomas Tharmabalan, UKM).

Table 1 shows the ethnobotanical information, consumption method, and alleged medicinal properties based on the semistructured interviews conducted with the Semai of the Orang Asli of the 4 WEPs featured in this study. *Sauropus androgynus* (L.) Merr. (see Figure 3), often known as “Pucuk manis,” is a perennial shrub native to Borneo that is frequently consumed as a leafy vegetable [21]. Other common English names include “sweet leaf” and “star gooseberry.” It belongs to the Phyllanthaceae family and can be found growing wild, although removing the tip to encourage multiplication is necessary and much faster than commercial production. Among the locals, this plant is known as “sayur manis,” “chekur manis,” and other names. It gets its name from the sweetness the leaves give after being cooked. Numerous studies have documented its effectiveness against diabetes, anemia, and obesity [22,23].

**TABLE 1:** Types of wild edible plants, species name, family name, parts used, and alleged medicinal properties

Local name	Species name	Family name	Parts used	Cooked/raw	Native/naturalized	Location (village)	Alleged medicinal properties
Sayur manis^1^	*Sauropus androgynus* (L.) Merr.	Phyllanthaceae	Young shoots, leaves, and flowers	Cooked	Native	1,2,3	Increase lactation in pregnant women, fever, and cough
Pucuk Manis^2^							
Pucuk Ubi	*Manihot esculenta Crantz*	Euphorbiaceae	Roots and young leaves	Cooked	Naturalized	1,2,3	Reduce tiredness and headache and induce labor
Saya^1^	*Diplazium esculentum* (Retz.) Sw.	Athyriaceae	Fronds	Raw and cooked	Native	1,2,3	Skin infection, fever, headache, diarrhea, recovery after childbirth, and respiratory problems
Sayang^2^							
Snegoh^3^	*Strobilanthes crispa* Blume	Acanthaceae	Leaves	Cooked	Naturalized	3	Diabetes, a diuretic agent, improves the immune system and treats wounds

1 Village 1 refers to *Sauropus androgynus* (L.) Merr. as sayur manis and *Diplazium esculentum (Retz.) Sw*.as saya

2 Village 2 refers to *Sauropus androgynus* (L.) Merr. as pucuk manis and *Diplazium esculentum (Retz.) Sw*. as sayang

3 Village 3 refers to *Strobilanthes crispa Blume* as snegoh.

**FIGURE 4:**
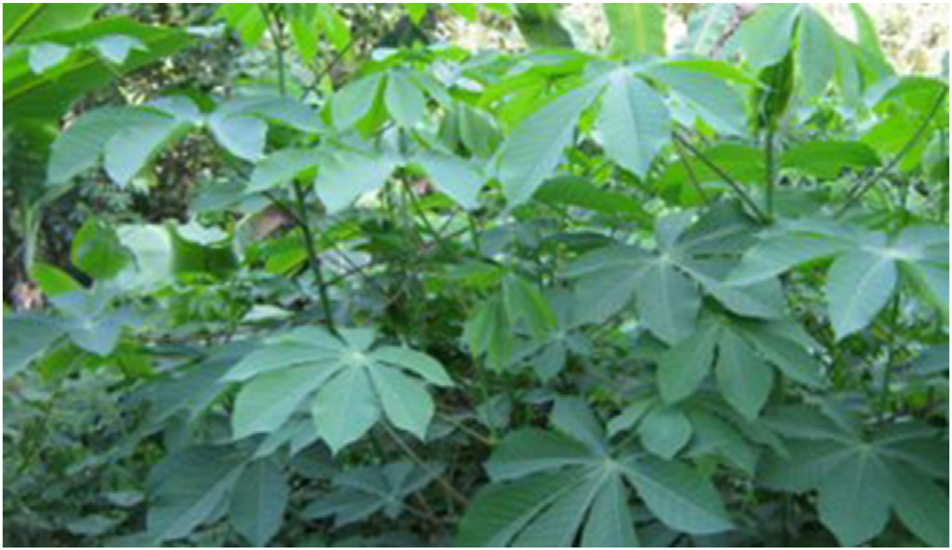
*Manihot esculenta Crantz* (Rachel Thomas Tharmabalan, UKM).

Cassava, or *Manihot esculenta Crantz* (see Figure 4), was introduced to Southeast Asia by colonial powers in the mid-nineteenth century primarily for the manufacture of starch and pearl tapioca [24]. It is a staple for 800 million people globally and is native to Amazonia [25]. *Manihot esculenta* is a Semai staple that needs to grow roughly 8–10 mo until harvestable. Because *Manihot esculenta* storage roots do not regenerate, stem cutting is used to proliferate them. The Semai community prefers to consume it cooked, steamed, or fried rather than raw. Before eating, the root is peeled, roasted, chopped, or dried to reduce the amount of cyanogenic glucosides present. On the other hand, the leaves have a far higher concentration of cyanogenic glucosides than the roots, and sun drying the leaves reduces the concentration to 90% of its original value [26].

**FIGURE 5:**
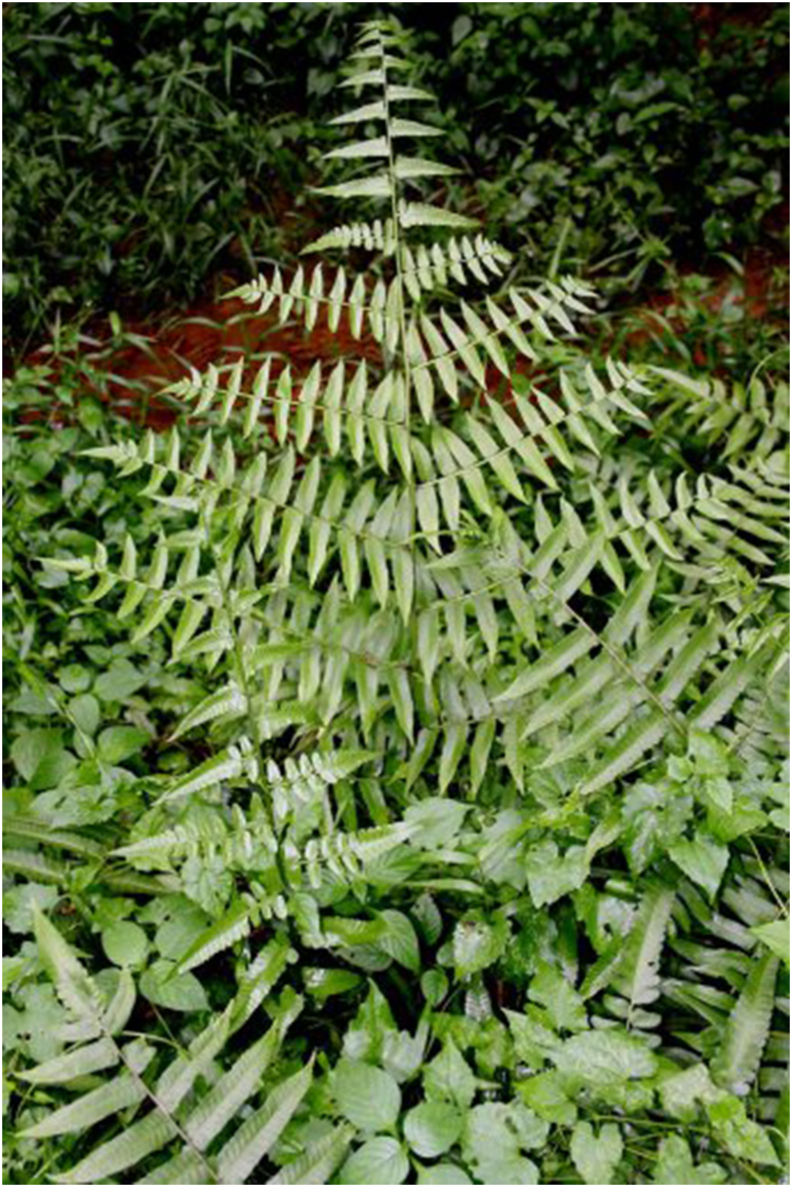
*Strobilanthes crispa* (Rachel Thomas Tharmabalan, UKM).

The perennial fern Diplazium esculentum (Retz.) Sw. (see Figure 5) also known as “fiddlehead” in English, is native to Asia and Oceania. They are members of the Athyriaceae family and thrive near riverbanks, especially during the wet season. It is known among the Semai as “saya” and “sayang,” as well as other local names such as “pucuk paku.” The fronds can be eaten raw or fried, and they are commonly utilized as a tonic by the Semai after childbirth [27]. It does not thrive in cooler climates and can become invasive in nature if heat and water are abundant. This fern is also utilized in traditional societies to treat tumors, asthma, and skin conditions [28].

**FIGURE 6:**
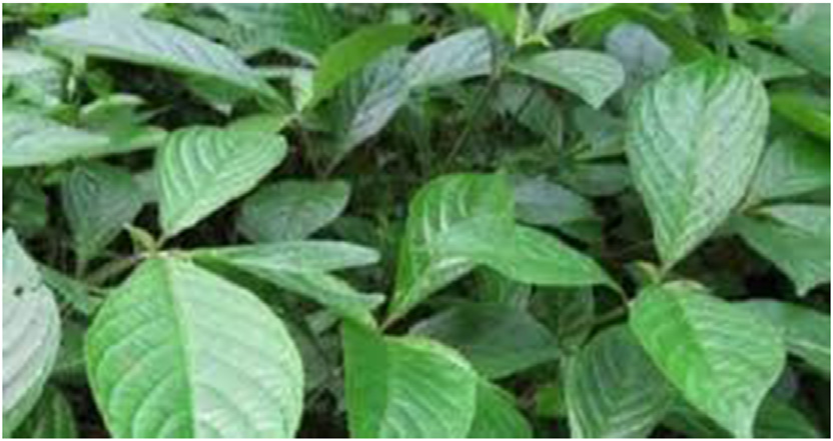
*Diplazium esculentum* (Retz.) Sw. (Rachel Thomas Tharmabalan, UKM).

*Strobilanthes crispus* Blume (see Figure 6), also called “snegoh” or “bern-go” among the Semai, and “black face general” in English, is a member of the Acanthaceae family that grows wild in Madagascar and Southeast Asia [29]. The flowers are yellow, short, and heavy, and the leaves of this plant are boiled and are usually drank as tea or infused water as it contains high minerals, polyphenols, and vitamin content [30]. *Strobilanthes crispus* leaves are grown in Malaysia for their usage in the pharmaceutical sector as a product that promotes health and for its traditional use as a diuretic, a blood thinner, and a treatment of cancer [31]. According to recent studies, the medicinal chemicals in the leaves may be a source of anticarcinogens and are, therefore, utilized as chemotherapeutic agents to treat cancer [32].

### Proximate composition

The proximate and mineral analyses of the 4 WEPs consumed by the Semai of the Orang Asli and 3 local commercial vegetables widely used among Malaysians are shown in Table 2. The 3 vegetables are *Ipomoea aquatica* (Kangkung), *Brassica rapa* subsp. *chinensis* (Pak Choy) and *Brassica oleracea var. alboglabra* (Kai Lan) were included in the table to allow for a comparison of nutrient and mineral content between traditional and commercially available vegetables [33]. The WEPs moisture composition ranged from 70.8 to 90 g/100 g, with the lowest being in *Manihot esculenta Crantz* and the highest in *Diplazium* esculentum (Retz.) Sw. The ash content ranged from 3.2 to 7.7 g/100 g and was much higher when compared to the selected 3 local commercial vegetables commonly consumed among Malaysians, ranging from 0.5 to 1.6 g/100 g. The WEPs reported in this study have much higher ash than commercial vegetables, ranging from 0.4% to 2.0% [34]. The fiber content found in *Strobilanthes crispa* Blume was 4.6 g/100 g, and the lowest was in *Sauropus androgynus*, whereas the fiber content for the commercial vegetables ranged from 1 to 2.6 g/100 g. The fiber content in all WEP vegetables was comparatively high compared to the 3 commonly consumed vegetables among Malaysians. Fiber-rich foods are necessary for proper digestion and waste disposal. Fibers can aid in lowering cholesterol concentrations as well as risk of obesity and coronary artery disease, diabetes, diverticulosis, colon and breast cancer, and hypertension [35,36]. The protein content for WEP vegetables ranged from 2.9 g/100 g to 7.2 g/100 g, with the highest being in *Diplazium esculentum* compared to the 3 selected local commercial vegetables, which had significantly lower protein content and ranged from 1.2 to 2.6 g/100 g*.* In a study conducted by Zanariah [37] on Malaysian leafy green vegetables, the protein content reported was much lower in a range of 0.8% to 1.6%. As protein deficiency is 1 of the contributing factors to malnutrition, the quantity and quality of the protein must be taken into consideration when considering the relative potential of WEPs as potential vegetables.

**TABLE 2:** Proximate and mineral analysis of 4 wild edible plants and 3 selected local commercial vegetables

Wild edible plants					Commercial vegetables		
Species name	*Sauropus androgynus* (L.) Merr.	*Manihot esculenta Crantz*	*Diplazium esculentum* (Retz.) Sw.	*Strobilanthes crispa* Blume	*Ipomoea aquatica*^1^	*Brassica rapa subsp. Hinensis*^1^	*Brassica oleracea var. Alboglabra*^1^
Moisture (g/100 g)	73.3 ± 0.75^2^	70.8 ± 0.12^2^	90 ± 0.14^2^	81.6 ± 0.53^2^	92.5	95.3	92.6
Ash (g/100 g)	6.85 ± 0.42^2^	3.2 ± 0.12^2^	7.7 ± 0.25^2^	4.2 ± 0.36^2^	1.6	0.8	0.6
Fiber (g/100 g)	2.39 ± 0.11^2^	3.7 ± 0.43^2^	3.2 ± 0.54^2^	4.6 ± 0.22^2^	2.1	1	2.6
Fat (g/100 g)	1.2 ± 0.22^2^	0.25 ± 0.11^2^	<0.1	<0.1	0.2	0.2	0.76
Protein (g/100 g)	6.9 ± 0.46^2^	5.7 ± 0.23^2^	7.2 ± 0.58^2^	2.9 ± 0.14^2^	2.6	1.5	1.2
CHO (g/100 g)	5.8 ± 0.14^2^	6.2 ± 0.52^2^	1.5 ± 0.14^2^	3.5 ± 0.29^2^	3.13	2.18	4.67
Energy (kcal/100 g)	61.6 ± 0.13	49.85 ± 0.22	34.8 ± 0.45	25.6 ± 0.34	19	13	26
Calcium (mg/100 g)	196 ± 1.21^2^	243 ± 0.76^2^	154 ± 0.35^2^	176 ± 0.59^2^	77	105	105
Iron (mg/100 g)	2.8 ± 0.21^2^	2.7 ± 0.08^2^	1.7 ± 0.11^2^	0.7 ± 0.03^2^	1.67	0.8	0.59
Potassium (mg/100 g)	498 ± 0.48^2^	527 ± 1.21^2^	515 ± 0.98^2^	295 ± 0.53^2^	312	252	274
Magnesium (mg/100 g)	63.8 ± 0.23^2^	97 ± 0.45^2^	37 ± 0.18^2^	32 ± 0.21^2^	71	19	19

The mean and standard deviation of triplicates (*n* = 3) was used to calculate the values at P <0.05.

1 Data obtained for the 3 local commercial vegetables were from USFDA [33].

2 Superscript showed that a significant difference exists: moisture, ash, fiber, fat protein, carbohydrate, calcium, iron, potassium, magnesium.

The caloric value for the WEPs selected ranged from 25.6 to 61.6 kcal/100 g, and for the 3 selected local commercial vegetables, it ranged from 13 to 26 kcal/100 g. The highest energy content was found in *Sauropus androgynus* and can be attributed to the higher carbohydrate content found in that vegetable. Similar to the results of the present investigation, Narzary et al. [38] investigated the energy content utilizing the same method used in this study to the value of various WEPs consumed by the Bodos, the early settlers of Assam, India and found that it ranged from 29.48 to 67.42 kcal/100 g of fresh sample. High-energy foods can be seen as part of a healthy human diet. These foraged edible plants with a high-calorie content can be used to create additional dietary supplements.

### Mineral composition

In terms of the mineral content in these wild edibles, the calcium content ranged from 154 to 243 mg/100 g. The plants are rich in calcium and arranged based on their calcium content in an ascending sequence: *Diplazium esculentum* < *Strobilanthes crispa* < *Sauropus androgynus* < *Manihot esculenta.* The WEPs investigated had higher calcium content than commercialized leafy greens, including *Ipomoea aquatica, Brassica rapa* subsp. *chinensis* and *Brassica oleracea var. Alboglabra* which ranged from 77 to 105 mg/100 g. Calcium is essential for the development of bone, the maintenance of teeth, and the contraction of muscles. It is also important for blood coagulation, cell permeability modulation, toxin removal, and nerve transmission [39,40]. The values obtained from this study suggest that WEPs may be a superior calcium supplier to several commonly consumed vegetables. Also, there is a calcium deficit of 45.08% among the Semai community. The current recommended nutrient intake for calcium is 400 mg/100 g, and these wild edibles could help supplement their calcium intake.

The potassium content recorded in this study ranged from 295 to 527 mg/100g. Three of the 4 WEP vegetables except for *Strobilanthes crispa* Blume exceeded the daily value (DV) and ranged between 10.6% and 11.2%. The link between high potassium intake and a reduced risk of hypertension and stroke has been highlighted by Williams [41] and a reduced risk of the formation of kidney stones [42]. Potassium is essential in ensuring one’s blood pressure is in a normal range as well as maintaining the electric conductivity of the brain and assisting in muscle contraction [43]. Recent studies show that the Orang Asli community has a prevalence of hypertension of 30.8%, which is 1.4% lower than the 3rd National Health Morbidity Survey [44]; as such, these WEPs could be an excellent dietary source to improve their potassium intake.

*Sauropus androgynus* contained 2.8 mg/100 g of iron (DV of 35% for males and 15.5% for female), comparable with *Manihot esculenta,* which contained 2.7 mg/100 g of iron (DV of 33.75% for males and 15% for females). In comparison to the 3 selected local commercial vegetables, the iron content ranged from 0.59 to 1.67 mg/100 g (DV of 7.4% to 20.9% for men and 3.3% to 9.3% for women). Iron is required to form hemoglobin, appropriate central nervous system function, oxygen transport, immunological function, and the oxidation of carbohydrates, proteins, and fats [45]. Anemia is caused by an iron deficiency and affects 24.6% of Malaysians aged 15 and above [46]. The PEANUTS study found that anemia and iron deficiency were present in 6.6% and 4.4% of Malaysian preschool and school-aged children, respectively [47]. The Semai community has a deficit of 59.30% of iron content [48]. Although the iron content is generally lower than the DV of 8 mg for males and 18 mg for females, the US Department of Agriculture [49] reports that anything that falls in the range of 10% to 19% of the DV makes it a good source, and anything above 20% is an excellent source. Furthermore, all these wild edibles can be considered a good source of iron as they fall in the range of 8.75% to 35% for men and 3.9% to 15.6% for women, with *Strobilanthes crispa* being on the lower end of the range.

The magnesium content in *Manihot esculenta* is the highest at 97 mg/100 g compared to other wild edibles and almost 6 times higher than *Strobilanthes crispa* (32 mg/100 g). For the 3 selected local commercial vegetables, the magnesium content ranged from 19 to 71 mg/100 g. Many enzymatic activities, glucose metabolism, and insulin homeostasis require magnesium as a cofactor [50]. According to Piuri et al. [51], a deficiency in magnesium is associated with obesity, metabolic syndrome risk, and type 2 diabetes.

These WEPs have demonstrated their effectiveness in nutritional studies because they contain the necessary micronutrients to help fight various non-communicable diseases. Studies done by many other researchers can also confirm this. For example, according to Nahak and Sahu [52], the leaves of *Sauropus androgynus* possess high antioxidant activity, which can be attributed to their high vitamins C and E content. Also, its protein content and affordability have elevated its status to multigreen or multivitamins [53]. Minerals such as Ca, Mg, Fe, Mn, Zn, and vitamins A and C can be found in the leaves of *Manihot esculenta* [54]. Hence, it can be an alternative food source to cassava’s primary consumers, who are usually from underdeveloped and developing countries, due to its low production cost and ability to withstand climate change [55,56]. However, the amount of *Manihot esculenta* leaves consumed can obstruct digestion and the absorption of nutrients due to toxic and antinutritional compounds found, such as trypsin inhibitors, tannin, phytate [57].

Research studies have shown that *Manihot esculenta* tuber cyanide can range from 75 to 1000 mg/kg, whereas its leaves can have between 1000 and 2000 milligram cyanogenic glycosides per kilogram of dry matter depending on the age of the plant, soil conditions, fertilizers, and environment [58,59]. Individuals who consume a diet high in cassava-based foods may experience health issues such as acute intoxications, chronic toxicity, neurological abnormalities, growth retardation, and goiter due to the presence of cyanogens in poorly processed food products [60].

Bender and Ismail [61] carried out the initial investigation on the dangers posed by *Sauropus androgynus*. The study showed that the fresh leaves of *Sauropus androgynus* contain 580 mg alkaloid “papaverine” per 100 g, which exceeds the 200 mg papaverine per day that is required as an antispasmodic drug. The tannin and oxalic acid content found in the leaves of *Sauropus androgynus* was 88.68 mg/100 g fresh weight (FW) and 33.25 mg/100 g FW respectively [53,62]. The unnecessary consumption of this plant by the elderly in Malaysia resulted in drowsiness and constipation. After *Sauropus androgynus* was launched as a supplement to lose weight in 1994, excessive intake and its effects first appeared in Taiwan in 1995 [63]. Following the consumption of *Sauropus androgynus*, cases of breathing difficulties, irreversible respiratory failure, and death have been documented. Constrictive bronchiolitis obliterans and other diseases were identified in those individuals’ histopathological examinations, and lung transplantation is the only way to treat the problem [64]. As such, these wild foods can subsequently be used to encourage consumption and aid the Semai of the Orang Asli community’s health and nutritional situation after undergoing additional pharmacological and toxicity research.

### Suitability of WEPs as mainstream crops

According to studies done by Dhanapal [49], there is a 58.33% deficit of green leafy and other vegetables among the Semai. By the time they reach the age of 5 or 6, malnutrition plays a significant role in their developmental outcomes as they only consume 3 servings of the recommended nutrient intake. In a study conducted by Cordelia [60], the Semai children did not meet the requirements of iron, riboflavin, vitamin C, folate, calcium, and thiamine in preschool children. All the vegetables highlighted in this study are available all year round and grow freely in the jungle close to the rainforest or along the riverbanks. As such it would be highly effective to recognize the potential of these WEPs to combat the DBM and enhance food and nutritional security.

In conclusion, this research shows that all 4 WEPs have variable quantities of proximate composition and mineral composition. These WEPs show higher or comparable values with common domesticated species here in Malaysia. *Manihot esculanta* had the highest carbohydrate, calcium, potassium, and magnesium content, whereas the highest ash and protein content was found in *Diplazium esculentum*. According to the current study’s findings, these wild plants can be regarded as inexpensive and good sources of micronutrients necessary for health and well-being. They can also contribute substantially to dietary needs, especially in remote areas, because of their beneficial nutritional qualities. Before they may be marketed as novel vegetables, more research is needed to evaluate the antinutritional elements and mineral bioavailability.

## Data Availability

Data described in the manuscript, code book, and analytic code will be made publicly and freely available without restriction at https://osf.io/g7psk/

## References

[1] Gosden C., Hather J.G. (2004).

[2] Bhatia H., Sharma Y.P., Manhas R.K., Kumar K. (2018). Traditionally used wild edible plants of district Udhampur, J&K, India. J. Ethnobiol. Ethnomed..

[3] Maduka S.M., Chikamai B., Eyog-Matig O., Mbogga M. (2004). Review and Appraisal on the Status of Indigenous Fruits in Eastern Africa, Synthesis Report.

[4] Della A., Paraskeva-Hadjichambi D., Hadjichambis A.C. (2006). An ethnobotanical survey of wild edible plants of Paphos and Larnaca countryside of Cyprus. J. Ethnobiol. Ethnomed..

[5] Bellwood P., Shuhaimi N.H. (1998). The Encyclopedia of Malaysia.

[6] (2019). Children, food and nutrition: state of the world’s children 2019: a look at child malnutrition in Malaysia and beyond. [Internet].

[7] (2019). Malaysia and WHO call for more investment in primary health care in the 21st century [Internet].

[8] Shrimpton R., Rokx C. (2012). http://documents.worldbank.org/curated/en/905651468339879888/The-double-burden-of-malnutrition-a-review-of-global-evidence.

[9] Kennedy G., Nantel G., Shetty P. (2003). The scourge of hidden hunger: global dimensions of micronutrient deficiencies. Food Nutr. Agric..

[10] (2016). Significant findings of forest biodiversity scientific expeditions [Internet].

[11] Humphrey C. (2019). Indigenous communities, nat’l parks suffer as Malaysia razes its reserves.

[12] (1996). Malaysia: country report to the Food and Agriculture Organization. Technical conference on plant genetic resources [Internet].

[13] Pieroni A. (2021). Wild foods: a topic for food pre-history and history or a crucial component of future sustainable and just food systems?. Foods.

[14] Khalid N.M., Hamid A.Q.A., Ramakrishna C., Cheng I., Chong Z.Y., Makbul I.A.A. (2017). Effects of selected herbs and vegetables on the nutritional quality of beef burger and rat bioassay. Sains Malays.

[15] You Y.X., Shahar S., Haron H., Yahya H.M. (2018). More ulam for your brain: a review on the potential role of ulam in protecting against cognitive decline. Sains Malays.

[16] (2021). Find & use location coordinates.

[17] (2022). Malaysia Australia Free Trade Area MAFTA world map.

[18] Martin G.J. (1995).

[19] ISE Ethics Toolkit [Internet] (2020). https://www.ethnobiology.net/what-we-do/core-programs/ise-ethics-program/ethics-toolkit/.

[20] (2000). Official methods of analysis of the Association of Official Analytical Chemists.

[21] Petrus A.J.A. (2013). *Sauropus androgynus* (L.) Merrill-a potentially nutritive functional leafy-vegetable. Asian J. Chem..

[22] Suparmi S., Nur A.C., Alvenia M.E., Galuh D.U., Iqrommatu I.L., Heavin R.S. (2016). Anti-anemia effect of chlorophyll from katuk (Sauropus androgynus) leaves on female mice induced sodium nitrite. Pharmacogn. J..

[23] Warditiani N.K., Milawati N.M.P. (2016). Susanti, Anti dyslipidemic activity of katuk leaves saponins fractions (*Sauropus androgynes* (L) Merr) in rats induced with fat-rich diet. Int. J. Pharm. Pharm. Sci..

[24] However R.H., Setiawan A., Fuglie K.O. (2005). Sweet Potato Research and Development: Its Contribution to the Asian Food Economy.

[25] Save and grow (2013). http://www.fao.org/3/a-i3278e.pdf.

[26] Wanapat M. (2009). Potential uses of local feed resources for ruminants. Trop. Anim. Health Prod..

[27] Bidin A. (1985).

[28] Zannah F., Amin M., Suwono H., Lukiati B. (2017). Phytochemical screening of *Diplazium esculentum* as medicinal plant from Central Kalimantan, Indonesia. AJP Conference Proceedings.

[29] Sunarto P.A. (1977).

[30] Ismail M., Manickam E., Danial A.M., Rahmat A., Yahaya A. (2000). Chemical composition and antioxidant activity of *Strobilanthes crispus* leaf extract. J. Nutr. Biochem..

[31] Yap L.S., Lee W.L., Ting A.S.Y., Agrawal D.C., Tsay H.S., Shyur L.F., Wu Y.C., Wang S.Y. (2017). Medicinal Plants and Fungi: Recent Advances in Research and Development.

[32] Ng M.G., Ng C.H., Ng K.Y., Chye S.M., Ling A.P.K., Koh R.Y. (2021). Anticancer properties of*Strobilanthes crispus*: a review. Processes.

[33] Food and Drug Administration (2016). Food labeling: revision of the nutrition and supplement facts labels, Fed. Regis.

[34] Roe M., Church S., Pinchen H., Finglas P. (2013).

[35] Roberfroid M. (1993). Dietary fiber, inulin, and oligofructose: a review comparing their physiological effects. Crit. Rev. Food Sci. Nutr..

[36] Spiller G. (2001).

[37] Zanariah J., Rehan A.N., Rosmah O. (1986). Protein and amino acid composition of Malaysian vegetables. MARDI Res. Bull..

[38] Narzary H., Swargiary A., Basumatary S. (2015). Proximate and vitamin C analysis of wild edible plants consumed by Bodos of Assam, India. J. Mol. Pathophysiol..

[39] Indrayan A.K., Sharma S., Durgapal D., Kumar N., Kumar M. (2000). Determination of nutritive value and analysis of mineral elements for some medicinally evaluated plants from Uttaranchal. Curr. Sci..

[40] Ghani A., Ali Z., Ishtiaq M., Maqbool M., Parveen S. (2012). Estimation of macro and micronutrients in some important medicinal plants of Soon Valley, District Khushab, Pakistan. Afr. J. Biotechnol..

[41] Williams D.E. (1993). Lycianthes moziniana (Solanaceae): an underutilized Mexican food plant with “new” crop potential. Econ. Bot..

[42] Weaver C.M. (2013). Potassium and health. Adv. Nutr..

[43] Ascherio A., Rimm E.B., Hernán M.A., Giovannucci E.L., Kawachi I., Stampfer M.J. (1998). Intake of potassium, magnesium, calcium, and fiber and risk of stroke among US men. Circulation.

[44] Darwina N., Wan Puteh S.E. (2012). Burden of non-communicable diseases among the Orang Asli community and patient satisfaction on non-communicable diseases management at public health facilities. BMC Public Health.

[45] Insel P., Ross D., McMahon K., Bernstein M. (2011).

[46] Poh B.K., Ng B.K., Siti Haslinda M.D., Nik Shanita S., Wong J.E., Budin S.B. (2013). Nutritional status and dietary intakes of children aged 6 months to 12 years: findings of the Nutrition Survey of Malaysian Children (SEANUTS Malaysia). Br. J. Nutr..

[47] Institute for Public Health (IPH) (2015).

[48] United States Department of Agriculture (2015). https://ndb.nal.usda.gov/ndb/.

[49] Dhanapal A.C.T.A., Subapriya M.S., Aung H.P. (2018). Household food and nutrient intake of Semai Aborigines of Peninsular Malaysia. Indian J. Sci. Technol..

[50] Song Y., Manson J.E., Buring J.E., Liu S. (2004). Dietary magnesium intake in relation to plasma insulin levels and risk of type 2 diabetes in women. Diabetes Care.

[51] Piuri G., Zocchi M., Della Porta M., Ficara V., Manoni M., Zuccotti G.V. (2021). Magnesium in obesity, metabolic syndrome, and type 2 diabetes. Nutrients.

[52] Nahak G., Sahu R.K. (2010). Free radical scavenging activity of multivitamin plant (*Sauropus androgynus* L. Merr.). Researcher.

[53] Singh S., Singh D.R., Salim K.M., Srivastava A., Singh L.B., Srivastava R.C. (2011). Estimation of proximate composition, micronutrients and phytochemical compounds in traditional vegetables from Andaman and Nicobar Islands. Int. J. Food Sci. Nutr..

[54] Ravindran V., Machin D., Nyvold S. (1992). Roots, Tubers, Plantains and Bananas in Animal Feeding.

[55] Ravindran V., Rajaguru A.S.B. (1988). Effect of stem pruning on cassava root yield and leaf growth. J. Agric. Sci..

[56] (2016). https://www.oecd-ilibrary.org/docserveid=id%26accname=guest%26checksum=BDCF28BA0699C.

[57] Jamil S.S., Bujang A. (2016). Nutrient and antinutrient composition of different variety of Cassava (Manihot esculenta Crantz) leaves. J. Teknol..

[58] Imakumbili M.L.E., Semu E., Semoka J.M.R., Abass A., Mkamilo G. (2019). Soil nutrient adequacy for optimal cassava growth, implications on cyanogenic glucoside production: a case of konzo-affected Mtwara region, Tanzania. PLOS ONE.

[59] (2004). Hydrogen cyanide and cyanides: human health Aspects [Internet].

[60] Nyirenda K.K., Erkekoglu P., Ogawa T. (2020). Toxicity potential of cyanogenic glycosides in edible plants.

[61] Bender A.E., Ismail K.S. (1973). Nutritive value and toxicity of Sauropus androgynous. Proc. Nutr. Soc..

[62] Sheela K., Nath K.G., Vijayalakshmi D., Yankanchi G.M., Patil R.B. (2004). Proximate composition of underutilized green leafy vegetables in Southern Karnataka. J. Hum. Ecol..

[63] Lai R.S., Chiang A.A., Wu M.T., Wang J.S., Lai N.S., Lu J.Y. (1996). Outbreak of bronchiolitis obliterans associated with consumption of *Sauropus androgynus* in Taiwan. Lancet.

[64] Yamamoto M., Higashimoto I., Oonakahara K., Watanabe M., Machida K., Matsuyama W. (2004). Clinical feature of bronchiolitis obliterans associated with consumption of Sauropus androgynus. Jpn. J. Chest Dis..

